# Physical Therapist-Led Therapeutic Exercise and Mobility in Adult Intensive Care Units: A Scoping Review of Operational Definitions, Dose Progression, Safety, and Documentation

**DOI:** 10.3390/jcm14248948

**Published:** 2025-12-18

**Authors:** Kyeongbong Lee

**Affiliations:** Department of Physical Therapy, Kangwon National University, Samcheok 25949, Republic of Korea; kblee@kangwon.ac.kr

**Keywords:** physical therapists, exercise therapy, mobility limitation, early ambulation, outcome assessment, health care, intensive care units

## Abstract

**Background/Objectives**: Intensive care units (ICU) immobility and weakness impair recovery, yet practice for Physical Therapist (PT)-led therapeutic exercise and mobility varies in definitions, dosing, safety, and documentation, which limits comparability and complicates quality assessment. This study aims to integrate adult ICU evidence and present PT-led operational definitions, dose progression principles, safety parameters, outcome measurement, and a documentation minimum dataset. **Methods**: A scoping review following PRISMA-ScR is used. Eligibility used Population, Concept, and Context: adults in ICU; PT-led therapeutic exercise or mobility; and ICU-initiated or directed care. Primary studies and prespecified quality-improvement reports were included. Data were extracted with a standardized form and summarized descriptively without meta-analysis. **Results:** Sixty studies were included. Based on the extracted data, this review synthesizes current evidence to propose standardized PT-led operational definitions and a graded progression from in-bed exercise to ambulation. While the individual components are derived from the literature, the conceptual framework for safety parameters and the stop rules were integrated and elaborated to guide clinical decision-making. Adverse events were uncommon and manageable. Outcome measurement centered on validated mobility and function measures at prespecified time points. A concise electronic record minimum dataset specifies provider attribution, timing and duration, activity level with assistance or device, planned and delivered dose with progression, in-session responses, and adverse events, supporting unit-level quality review and comparisons across ICU. **Conclusions**: A PT-led, graded program that applies shared thresholds, uses validated outcome measures, and employs standardized electronic documentation is feasible and supports safe delivery, clinically meaningful change, and quality improvement across adult ICU.

## 1. Introduction

Recent guidance emphasizes structured rehabilitation for critically ill adults and treats immobility as a core domain of intensive care units (ICU) care [1]. The 2025 update recommends enhanced mobilization or rehabilitation in addition to usual care, supporting protocol-driven, team-based practice [2]. Within structured ICU rehabilitation, physical therapist (PT)-led exercise and mobility are prescribed and documented as part of routine care.

ICU rehabilitation is multidisciplinary, including PT, occupational therapy (OT), cognitive rehabilitation, and dysphagia management [3,4]. This review focuses on PT-led therapeutic exercise and mobility, including assessment, dose progression, safety criteria, and documentation, embedded in a multidisciplinary program so that discipline-specific roles are clearly defined and integration with nursing, respiratory therapy, and medical teams is maintained [5,6,7]. Clarifying provider attribution and documentation fields is essential for consistent delivery, quality improvement, and reimbursement readiness.

Initiating PT and OT during mechanical ventilation was associated with improved functional outcomes and mobility targets, and program evaluations reported positive effects on process measures and length of stay (LOS), with variable effects on mortality [8,9,10,11]. Inconsistent findings likely reflect heterogeneity in definitions, dose, timing, and fidelity. In parallel, feasibility and safety studies indicated low adverse event rates when mobilization follows standardized screening and stop rules [12,13,14]. Taken together, the evidence supports standardizing operational definitions and role attribution, outcome measures and time points, frequency/intensity/time/type (FITT)-based dosing and progression, safety screening with stop rules, and documentation fields with unit-level indicators [15,16,17,18].

To inform clinical decisions and support quality assessment, validated and feasible assessments for the ICU are used, including the ICU Mobility Scale (IMS), the Functional Status Score for the ICU (FSS-ICU), and the Medical Research Council (MRC) sum score. These outcome measures are administered at prespecified time points such as early baseline, a midpoint during the ICU stay, and ICU discharge [19,20,21,22,23,24]. Safety protocols are based on consensus criteria for mobilizing mechanically ventilated adults [12,25], and previous studies describe clear thresholds and stop rules associated with safe practice [13,14]. This alignment ensures that planned and delivered dose, intensity, progression decisions, reasons for session hold, and adverse events are consistently recorded and used to derive unit-level indicators [15,16,17,18].

Given variability in terminology, attribution, dose rules, safety criteria, and documentation, this scoping review describes current practice, identifies reporting inconsistencies, and informs implementation without estimating comparative effects [1,2]. This review is structured by key implementation components that reflect care delivery and include operational definitions and scope, provider attribution, outcome instruments and timing, FITT-based dosing and progression, safety screening and monitoring with stop rules, documentation fields for the electronic medical record (EMR), and unit-level quality indicators. By synthesizing current evidence to conceptually elaborate on PT-led assessment, dose and progression, safety procedures, and documentation within a multidisciplinary approach, this review constructs a unified framework that supports consistent clinical implementation, enables unit-level performance reports and between-site comparisons, and provides a structured documentation approach for reimbursement readiness across health systems.

## 2. Materials and Methods

### 2.1. Study Design and Reporting

This scoping review followed the Preferred Reporting Items for Systematic Reviews and Meta-Analyses extension for Scoping Reviews (PRISMA-ScR) [26]. Methods and reporting adhered to the Joanna Briggs Institute (JBI) guidance for scoping reviews [27]. No meta-analysis was planned and no unpublished data were used. An a priori protocol was developed and not registered. The protocol and the completed PRISMA-ScR checklist are provided in Supplementary S1 [28].

### 2.2. Information Sources and Search Strategy

Electronic databases, including PubMed, Embase, CINAHL, the Cochrane Central Register of Controlled Trials, Web of Science Core Collection, and KoreaMed, were searched from 1 January 2000 to 31 October 2025 [29]. Search filters were applied to restrict results to human subjects and publications in English or Korean. To ensure full reproducibility, the complete search strings for each database, including all Boolean operators and controlled vocabulary (e.g., MeSH), are provided in Supplement S1 [30]. ClinicalTrials.gov was also checked to identify ongoing studies, although eligibility was limited to peer-reviewed publications.

### 2.3. Eligibility Criteria

Eligible studies enrolled adults aged 18 years or older in the ICU and examined PT-led therapeutic exercise or mobility in which therapists were responsible for planning, dose prescription, progression, or delivery. Intervention content included in-bed therapeutic exercise, sitting activities, sit-to-stand, standing, ambulation in the ICU, and task-specific training. Study designs were prospective original research or prespecified quality-improvement reports, reported in English or Korean.

Exclusion criteria were pediatric or neonatal intensive care settings, interventions delivered outside the ICU, and unclear provider attribution. Routine turning, transport, or hygiene repositioning without therapeutic intent; biologic or pharmacologic uses of mobilization; non-peer-reviewed formats or conference abstracts; and case reports without extractable information on dose, progression, or safety were excluded. Records with insufficient information after full-text review and duplicate publications were also excluded.

### 2.4. Study Selection

Records were de-duplicated and screened by title and abstract against the eligibility criteria [29]. Search results were exported to a reference manager, duplicates were removed, and the reference lists of included studies were screened [30]. Screening prioritized records that clearly named physical therapy with ICU terms; records describing exercise or mobility in the ICU without clearly defined provider terms were retained when rehabilitative intent was evident and biologic uses of “mobilization” were absent. Full texts of potentially eligible records were assessed, exclusion reasons were coded using predefined categories, and unretrieved reports were noted. Screening and full-text assessment were undertaken by a single reviewer. Although independent double review was not feasible due to the study design, measures were implemented to minimize bias and ensure rigor. A random 20% sample at both stages was rechecked by the author after a predefined interval with original labels concealed to establish intra-rater reliability, and any discrepancies were resolved by reapplying protocol rules.

### 2.5. Data Charting and Items

Documentation was aligned with the Template for Intervention Description and Replication [15] and the Standards for Quality-Improvement Reporting Excellence [16]. Data were extracted with a piloted form covering study descriptors, provider attribution, intervention content, dose and progression, safety procedures, outcome measures with timing, EMR fields, and unit-level quality indicators. Study descriptors included country, setting, intensive care unit type, design, admission diagnoses, and mechanical ventilation status.

Provider attribution was recorded as PT-led when therapists planned exercise or mobility, prescribed dose, made progression decisions, or delivered the intervention; records with ambiguous wording but rehabilitative features were classified as provider unclear. Intervention content was grouped as in-bed therapeutic exercise, sitting, sit-to-stand, standing, ambulation, and task-specific training. Dose followed FITT, and intensity indicators were recorded when available. Progression criteria and session hold criteria were extracted as reported; when algorithms were provided, decision points were summarized to maintain internal consistency with dose and safety criteria.

Safety criteria were grouped as oxygenation and ventilation, hemodynamics, sedation and delirium, and device and line categories. Screening parameters, in-session thresholds, stop rules, and adverse events were extracted. Outcome measures and time points were extracted, with common measures including the ICU Mobility Scale, the Functional Status Score for the ICU, and the MRC sum score. Documentation fields were recorded when EMR charting was feasible, including planned and delivered dose, reasons for stopping, progression decisions, provider discipline, location, equipment, and adverse events. Intervention indicators, including time to first mobilization, highest mobility achieved, and the proportion of therapy-eligible days, were summarized descriptively. A single reviewer extracted data. A random 20% sample was re-extracted after a predefined interval with original entries concealed to minimize recall bias. Consistent with the aim of mapping operational definitions and safety parameters rather than assessing intervention efficacy, a formal critical appraisal of individual studies was not performed. This approach aligns with the PRISMA-ScR guidelines, which indicate that quality assessment is optional for scoping reviews [26].

### 2.6. Synthesis of Results

Evidence was summarized across predefined areas covering operational definitions and scope, provider attribution, outcome measures and timing, dose and progression, safety screening and monitoring with stop rules, and documentation fields with unit-level quality indicators. Specifically, the operational definitions and safety frameworks presented in the results represent a conceptual elaboration by the authors, synthesized from the included studies to bridge gaps where explicit definitions were absent or inconsistent in the literature. Detailed specifications are provided in structured tables and a documentation codebook. Provider attribution was stratified as PT-led or provider unclear and summarized separately. Safety parameters were standardized within this review and grouped as oxygenation and ventilation, hemodynamics, sedation and delirium, and device and line categories. Outcome measures were summarized by instrument and planned timing, and documentation fields were distinguished as described versus implemented in the EMR when available. Only primary studies and prespecified quality-improvement reports contributed to counts, while guidelines and reviews informed contextual interpretation.

## 3. Results

**Figure 1:**
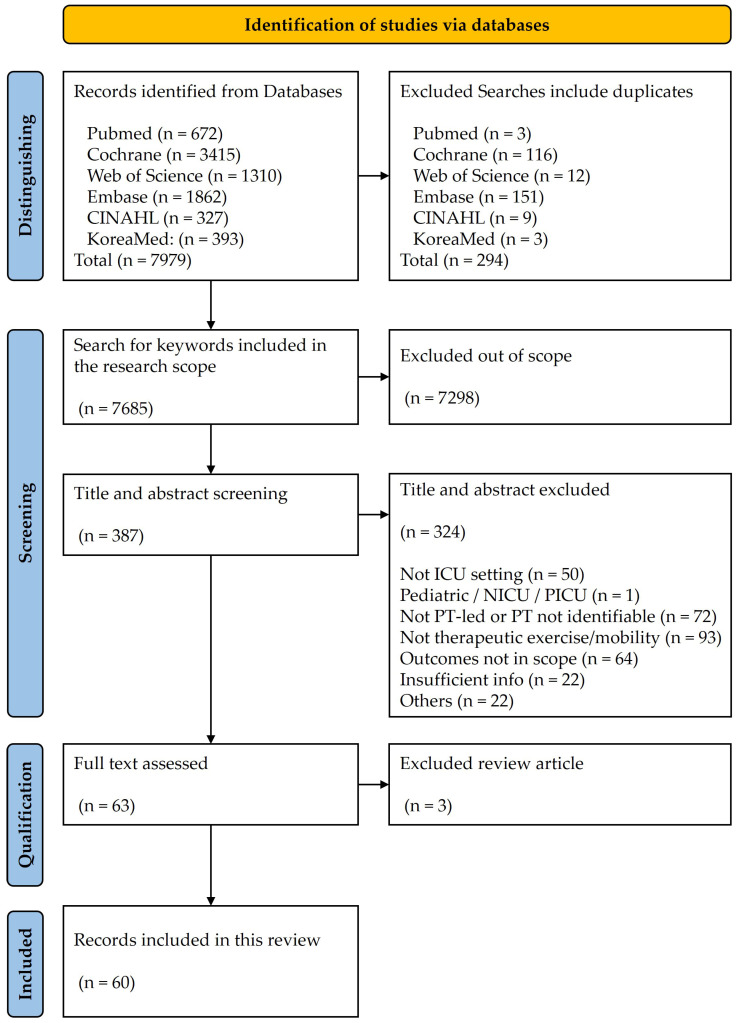
PRISMA-ScR flow diagram of study identification, screening, full-text eligibility assessment, and inclusion for PT-led therapeutic exercise and mobility in adult ICU.

Database searches identified 7979 records across PubMed, Cochrane, Web of Science, Embase, CINAHL, and KoreaMed. After removal of 294 duplicates, 7685 records remained and were filtered to the ICU rehabilitation scope, which excluded 7298 records as out of scope and left 387 for title and abstract screening. Title and abstract screening excluded 324 records for the following reasons: not ICU setting, pediatric or neonatal ICU focus, provider not identifiable as PT-led, not therapeutic exercise or mobility, outcomes not in scope, insufficient information, and other reasons. Full texts were assessed for 64 records; 3 reviews were excluded. Finally, 60 studies met eligibility and were included in the review. The flow of records is presented in Figure 1. Given the heterogeneity of the included studies, the extracted data were synthesized into structured operational frameworks to facilitate mapping. Table 1, Table 2, Table 3, Table 4, Table 5, Table 6 and Table 7 present these synthesized findings, systematically categorizing the evidence into PT-led operational definitions, provider attribution, outcome measures with planned time points, FITT-based dosing with graded progression, safety screening including stop rules, and the minimum EMR dataset.

Table 1 describes and defines PT-led activity categories are defined with clear inclusion and exclusion, and assessment, prescription, and progression are under therapist responsibility. In-bed therapeutic exercise emphasizes planned sets and repetitions to build tolerance, and routine positioning or hygiene-only repositioning is excluded. Sitting at the edge of the bed (EOB) targets postural control and orthostatic adaptation, and progression increases duration and balance demands. Sit-to-stand and static standing reduce assistance while repetitions and hold time increase, preparing for stepping. Bed-to-chair transfer specifies active pivot or stand-step with secure line and tube management, and transport-only moves are excluded. Ambulation increases distance and pace while assistance and device support decrease. Task-specific training addresses bed mobility, transfers, reaching, and dual-task practice aligned to discharge goals.

Table 2 summarizes PT-led device- and technology-assisted therapeutic exercise across modalities. Upper-limb ergometry is dosed by cadence or resistance. In-bed lower-limb cycle ergometry advances active time and cadence after a readiness screen. Body-weight support treadmill (BSWT) walking enables early gait using a harness and unloading, and progression increases speed while unloading is reduced. Robotic or suspension systems facilitate early standing or stepping under therapist control, and assistance is reduced as task demands increase. Neuromuscular electrical stimulation is used in conjunction with exercise with parameters set for pulse width, frequency, and duty cycle. Virtual reality or combined cognitive and physical modules function as complementary modules that enhance engagement across levels. Prescription variables include cadence, resistance, speed, and session minutes.

Table 3 demonstrates a PT-led model with explicit role attribution. Standardized orders or automatic consults enable early initiation, and a daily checklist identifies eligible patients. Pre-session checks confirm oxygenation, hemodynamics, arousal, and line security. Sessions proceed with light sedation targets and, when appropriate, on low-dose vasoactive support under enhanced monitoring. In-session monitoring includes Fraction of Inspired Oxygen (FiO_2_), positive end-expiratory pressure (PEEP), Peripheral Capillary Oxygen Saturation (SpO_2_), Heart Rate (HR), Mean Arterial Pressure (MAP), cardiac rhythm, and symptoms, with predefined stop rules and a plan to resume at a lower level once stable. The electronic medical record records provider, activity level, planned and delivered dose, and adverse events. Daily mobility rounds include the PT to align goals and progression across the team.

Table 4 summarizes outcome measures and timing focus on validated mobility and function assessments. The IMS and the FSS-ICU indicate mobility status, and the MRC sum score indicates strength. The Physical Function in Intensive Care Test (PFIT) scored and the 6-Minute Walk Test (6MWT) are used when feasible. Common time points include the first feasible ICU assessment, daily or ICU discharge, and, when possible, hospital discharge or short-term follow-up. Administration favors brief bedside testing with minimal equipment and alignment with the safety rules summarized in Table 6.

In Table 5, progression algorithms are presented within a PT-led model. Readiness addresses oxygenation, hemodynamics, light sedation, and secure lines. A consistent sequence is applied across activities. Time is extended first, task difficulty or intensity is adjusted next, assistance is reduced as tolerated, and then the activity level is advanced. This pattern is applied to in-bed exercise, EOB activities, transitions, transfers, ambulation, ergometry, treadmill or body-weight support, robotic or suspension assistance, and task-specific training.

The safety structure is organized as pre-session screening, in-session monitoring, and stop rules with actions (Table 6). Typical thresholds include a FiO_2_ of up to 0.6, SpO_2_ at least 90%, PEEP 10 to 12, MAP at least 65 mmHg, and RASS −2 to 0. Participation on low-dose vasoactive support is feasible with closer observation. During sessions, SpO_2_, HR, Blood Pressure (BP), cardiac rhythm, and symptoms are observed and the work rate or time is adjusted according to tolerance. Stop rules are triggered by hypoxemia, hypotension or other hemodynamic instability, arrhythmia, respiratory distress, reduced arousal or agitation, device or line compromise, new neurologic signs, or patient request. Actions include immediate pause, seated rest and stabilization, securing lines and devices, adjusting oxygen according to local protocol, documenting the event and response, and resuming at a lower activity level once stable.

Table 7 specifies the documentation minimum dataset which specifies EMR fields to implement and review PT-led sessions. Required entries include provider and co-attending staff, session start and end time with duration, pre-session safety parameters, activity level with assistance or device, planned and delivered dose with progression, in-session responses, and adverse events. Quality rules check completeness, internal consistency with activity level and device or assistance, alignment with safety thresholds from Table 6, and documentation of any event and response.

Supplement S2 defines the intervention range by level, covering in-bed, edge of bed, transition, transfer, and ambulation, with readiness criteria, typical dose parameters, minimum staffing and roles, and equipment with safety features. It provides a graded structure that links activity content with safety and delivery. Supplement S3 presents a concise documentation codebook that defines each EMR field, specifies field format and units or scales, clarifies documentation timing, indicates whether the field is required, and lists validation rules and allowed values so that data documentation is consistent and measurable. Supplement S4 synthesizes common barriers to ICU rehabilitation into concise categories and pairs each barrier with PT-led strategies, the key enablers and resources needed, and practical metrics to track implementation progress. Together these supplements operationalize how activities are delivered, how they are documented, and how unit-level obstacles are addressed to sustain PT-led practice. In addition, these supplements define how activities are delivered, how they are documented, and how unit-level obstacles are addressed to sustain PT-led practice. References used in Supplements S2–S4 align with the documentation fields in Table 7 and follow the manuscript’s numbering. All sources cited in the Supplements are included in the main reference list, and table entries that present titles only correspond to the same numbered references. The Discussion cites these sources using the same numbering to maintain consistency and avoid duplication.

**Table 1:** Operational definitions and scope of PT-led therapeutic exercise and mobility in adult ICU.

Term & Operational Definition	Inclusion/Exclusion	Activity Level	PT-Led Components
In-bed therapeutic exercise (active/active-assisted): Goal-directed limb/trunk exercises (supine/semi-recumbent) with planned sets-reps to build activity tolerance and neuromuscular activation [31,32,33,34].	Include: active/active-assisted exercises with dosing; Exclude: routine passive positioning or hygiene-only repositioning.	In-bed (level 1)	Assessment: ROM/MRC sum score; Prescription: sets-reps and rest; Progression: ↑ reps/resistance → sitting on EOB.
Sitting at EOB: Supported/unsupported sitting with postural control and orthostatic adaptation tasks [35,36,37,38,39].	Include: ≥1–2 min EOB tasks; Exclude: brief roll without therapeutic intent.	Sitting on EOB (level 2)	Assessment: orthostatic response; Prescription: minutes/sessions; Progression: ↑ duration/complexity → sit-to-stand.
Sit-to-stand and static standing: Task-specific transitions from sitting to standing and stand-hold with assistance as required [33,37,39,40,41].	Include: repetitions for endurance/strength; Exclude: passive hoist with no active effort.	OOB transition (level 3)	Assessment: hemodynamics/SpO_2_; Prescription: reps and rest; Progression: ↓ assist → marching/mini-squats.
Bed-to-chair transfer: Active transfer (pivot/slide/stand-step) with secure line/tube management [32,36,39,40,41,42].	Include: PT-guided OOB transfer; Exclude: transport-only transfers without exercise intent.	OOB transfer (level 4)	Assessment: line security/RASS; Prescription: daily transfer goal; Progression: ↓ assist devices → independence.
Ambulation/gait (assisted to independent): Progressive walking focusing on distance, speed, and safety with aids as needed [32,39,40,41,42,43,44,45].	Include: goal-based ambulation; Exclude: standing pivot only.	Ambulation (level 5)	Assessment: IMS/FSS-ICU gait parameters; Prescription: distance/time targets; Progression: ↑ distance/speed, ↓ assist.
Task-specific functional training: Bed mobility, transfers, reaching/handling, dual-tasking aligned with discharge goals [36,37,46,47,48].	Include: repeated practice of ADL-relevant tasks; Exclude: non-goal passive movements.	Across levels	Assessment: FSS-ICU/PFIT-s components; Prescription: task reps/sets; Progression: ↑ complexity/dual-task.

Note: Include only PT-led therapeutic exercise/mobility within ICU. Exclude routine turning, transport, and hygiene repositioning. Abbreviations: PT, Physical Therapists; ROM, range of motion; MRC, Medical Research Council; EOB, edge of bed; OOB, out of bed; SpO_2_, Peripheral Capillary Oxygen Saturation; RASS, Richmond Agitation–Sedation Scale; IMS, ICU Mobility Scale; FSS-ICU, Functional Status Score for ICU; ADL, activities of daily living; PFIT, Physical Function ICU Test. ↑, increase; ↓, decrease.

**Table 2:** PT-led device-/technology-assisted therapeutic exercise in adult ICUs.

Therapeutic Devices	Inclusion/Exclusion	Activity Level	PT-Led Components
Upper-limb ergometer exercise: Arm ergometry with set cadence/resistance for aerobic/strength goals [44,47,49].	Include: cadence/RPE-dosed sessions; Exclude: unplanned ROM only.	In-bed/EOB/OOB	Assessment: RPE/HR; Prescription: RPM/Watt/min; Progression: ↑ cadence/resistance.
In-bed cycle ergometry (lower limbs): Bedside leg cycling (active/assisted) with graded cadence and duration [31,47,50].	Include: protocolized cycling with safety screen; Exclude: CPM-type passive motion without rehab intent.	In-bed → OOB	Assessment: oxygenation/hemodynamics; Prescription: minutes and cadence; Progression: ↑ active time/gear.
Bedside treadmill/BWST: Harness-assisted treadmill stepping enabling early gait with unloading for safety [51,52].	Include: BWST with harness and team support; Exclude: unstable patients without safeguards.	OOB high-level	Assessment: orthostatic/line security; Prescription: speed/min; Progression: ↑ speed, ↓ unloading.
Robotic-/suspension-assisted mobilization: Robotic or suspension systems to facilitate early standing/stepping with controlled assistance [53,54].	Include: device use under PT control; Exclude: device-only passive movement without goals.	OOB assistive	Assessment: device fit/safety; Prescription: session min and task blocks; Progression: ↓ assistance, ↑ task demand.
NMES: Surface electrical stimulation adjunct to exercise to mitigate atrophy or prime muscles [55,56,57,58,59].	Include: NMES with therapeutic goals; Exclude: NMES alone as sole intervention without rehab plan.	Co-interventions to various exercises	Assessment: target muscle/parameters; Prescription: pulse width/frequency/on–off; Progression: ↑ intensity/duration.
VR or combined cognitive-physical (adjunct): VR or cognitive modules integrated with exercise to enhance engagement/dual-task capacity [46,60,61].	Include: interactive modules with goals; Exclude: passive viewing only.	Complementary modalities across levels	Assessment: cognitive tolerance; Prescription: minutes and difficulty; Progression: ↑ challenge/dual-task load.

Abbreviations: RPE, rating of perceived exertion; HR, Heart Rate; EOB, edge of bed; OOB, out of bed; RPM, revolutions per minute; CPM, continuous passive movement; BWST, body-weight supported training; NMES, neuromuscular electrical stimulation; VR, virtual reality. ↑, increase; ↓, decrease.

**Table 3:** Provider attribution and team roles for Physical Therapists-led therapeutic exercise and mobility in adult ICUs.

Operational Statement	PT	OT	RN	MD	RT	Co-Management Notes	Documentation Phrase Example
PT explicitly stated as prescriber/leader of therapeutic exercise and mobility [36,38,62,63].	✓			✓		Orders may be co-signed per policy.	Physical Therapist prescribed and progressed therapeutic exercise and mobility per protocol.
PT delivers intervention; leadership implied within interdisciplinary protocol [36,38,62,64].	✓		✓	✓	✓	Protocol defines roles; MD approves plan of care.	Mobility provided by PT within the interdisciplinary early rehabilitation protocol.
Standing order set or automatic PT consult within 24–48 h of ICU admission [32,36,38].	✓		✓	✓		RN triggers consult via protocol; MD approves; PT initiates assessment within timeframe.	Automatic PT consult triggered within 48 h of admission per ICU order set.
Algorithmic screening (e.g., daily checklist) identifies candidates for PT-led mobility [44,65].	✓		✓	✓	✓	Checklist covers hemodynamics, oxygenation, sedation, and lines; team confirms eligibility on rounds.	Daily mobility screen completed; patient cleared for PT-led session.
Target light sedation (e.g., RASS −1 to +1) to enable active participation [5,6,8,13,29].	✓		✓	✓		MD and RN titrate sedation; PT aligns timing/intensity.	RASS −1 to 0 prior to sit-to-stand; session intensity adjusted accordingly.
Mobility may proceed on low-dose vasoactive agents with enhanced monitoring and predefined stop rules [40,66,67].	✓		✓	✓		Dose thresholds and stability criteria defined; MD and RN confirm before session.	Mobilization performed on norepinephrine ≤ 0.1 µg/kg/min with continuous monitoring.
Line/tube security plan (ETT/tracheostomy, central/arterial lines, drains) agreed before mobilization [42,68,69,70].	✓		✓	✓	✓	RN secures lines; RT manages airway; PT leads movement plan.	All lines secured; RT present for ETT; PT leads transfer to chair.
Pre-session screen covers oxygenation (FiO_2_/SpO_2_/PEEP), hemodynamics (HR/MAP), sedation/delirium, and line security [31,46,69,70].	✓		✓	✓	✓	Team confirms parameters within acceptable ranges before starting.	Pre-session screen met: FiO_2_ ≤ 0.6, PEEP ≤ 10 cmH_2_O, MAP ≥ 65 mmHg.
In-session monitoring of SpO_2_, HR, BP, cardiac rhythm, and symptoms (dyspnea/Borg) [36,42,46,49].	✓		✓		✓	Telemetry/oximetry continuous; RT monitors ventilator parameters.	SpO_2_ and HR monitored continuously; Borg recorded each bout.
Terminate for hypoxemia/desaturation, arrhythmia, hypotension, neurologic change, or line compromise [36,40,44,46].	✓		✓	✓	✓	Predefined thresholds and response plan documented.	Session stopped for SpO_2_ < 88% or ↓ ≥4% from baseline; reassess and resume when stable.
EMR records the provider, activity level (e.g., IMS), planned vs. delivered dose, and adverse events [40,42,43,70].	✓	✓	✓			Standardized fields support audit and billing readiness.	PT: IMS = 6 (standing), planned 2 × 10 sit-to-stand; delivered 2 × 8; no adverse events.
Daily ICU mobility rounds include PT; goals updated and barriers addressed [32,37,43,68].	✓	✓	✓	✓	✓	Shared dashboard with unit indicators reviewed weekly/monthly.	Mobility goal updated to walk 10 m with assistive device; suction equipment arranged.

Abbreviations: PT, Physical Therapist; OT, Occupational Therapist; RN, Registered Nurse; MD, Medical Doctor; RT, Respiratory Therapist; ETT, Endotracheal Tube; FiO_2_, Fraction of Inspired Oxygen; PEEP, Positive End-Expiratory Pressure; SpO_2_, Peripheral Capillary Oxygen Saturation; HR, Heart Rate; MAP, Mean Arterial Pressure; IMS, ICU Mobility Scale. ✓, indicates involvement; ↓, decrease.

**Table 4:** Outcome instruments and measurement time points for PT-led adult ICUs rehabilitation.

Assessment/Outcome	Construct and Scoring	Common Measurement Time Points Observed	Interpretation and MCID/MDC	Administration and Feasibility
ICU Mobility Scale [35,36,38,40,41,62,65]	Eleven levels (0–10), higher = better; zero passive in-bed → ten independent ambulation.	ICU first feasible; daily; ICU discharge; sometimes hospital discharge or 30–90 day follow-up.	Higher = better; MCID/MDC not established in included studies.	PT/OT; ~1–2 min; no equipment; record highest level achieved; monitor SpO_2_/HR/BP per safety table.
Functional Status Score for the ICU [35,36,38,40,62,71]	Five mobility tasks (roll, transfer supine ↔ sit, sit ↔ stand, sit, walk); each 0–7; total 0–35, higher = better.	ICU first feasible; ICU discharge; often hospital discharge; sometimes 30–90-day follow-up.	Higher = better; MCID/MDC not established in included studies.	PT/OT; ~5–7 min; bed/chair, gait belt; monitor SpO_2_/HR/BP per safety table.
Medical Research Council sum score [31,40,53,55,62,70,72]	Six bilateral muscle groups 0–5; total 0–60, higher = better.	ICU first feasible; ICU discharge; often hospital discharge; sometimes 30–90-day follow-up.	Higher = better; MCID/MDC not established in included studies.	PT/OT; ~5–10 min; standardized positions; avoid excessive resistance if unstable; monitor per safety table.
Physical Function in ICU Test scored [40,62,64,71]	Sit-to-stand assistance 0–3, marching cadence 0–3, shoulder/knee strength 0–2; total 0–10, higher = better.	ICU first feasible; ICU discharge; occasionally hospital discharge or 30–90-day follow-up.	Higher = better; MCID/MDC not established in included studies.	PT/OT; ~5–7 min; chair, stopwatch/metronome for cadence; monitor per safety table.
6-Minute Walk Test [31,69,73]	Distance walked in 6 min (m); higher = better.	Hospital discharge or post-ICU follow-up when feasible.	MCID/MDC not established in included ICU studies; increase in meters indicates improvement.	PT/OT; measured corridor ~30 m; standardized protocol; monitor per safety table.
Other clinical outcomes (LOS, ventilator days, discharge destination, mortality) [36,37,38,40,42,62,65,71,74,75,76,77]	Service/clinical outcomes from EMR: ICU/hospital LOS (days), ventilator days, discharge destination, ICU/in-hospital mortality.	ICU discharge and hospital discharge; mortality also in-ICU/in-hospital and sometimes 30–90-day follow-up.	MCID/MDC not established; better outcomes correspond to fewer days, lower mortality, and discharge to home or inpatient rehabilitation.	Extract from EMR with predefined windows and definitions; note censoring and competing risks; align with safety table where relevant.

Abbreviations: MCID, minimal clinically important difference; MDC, minimally detectable change; LOS, length of stay.

**Table 5:** Physical therapists-prescribed progression algorithms by therapeutic exercises and modalities.

Parameters	Readiness Screen with Pass or Hold Cues	Typical Progression Sequence
In-bed therapeutic exercise (active/active-assisted) [37,38,78,79]	Stable oxygenation/hemodynamics; follows simple commands or assisted participation	Reps ↑ → task complexity ↑ → add light resistance → transition to EOB tasks
Sitting at EOB [32,35,36,37,38]	Orthostatic tolerance acceptable; lines secured; path clear	Duration ↑ → support ↓ → add balance tasks → prepare sit-to-stand
Sit-to-stand/static standing [32,33,37,40,42]	Orthostatic tolerance; lines secured; team spotter available	Assistance level ↓ → reps/stand time ↑ → add marching/weight-shift
Bed-to-chair transfer [32,36,37,42]	Stable oxygenation/hemodynamics; chair locked; airway/lines plan complete	Assistance level ↓ → transfer type advance (slide/pivot → stand-step) → rest ↓
Ambulation/gait [32,37,40,42,62,80]	Orthostatic tolerance; portable monitoring; lines secured with slack	Distance ↑ → pace ↑ → device support ↓ → dual-task/turns ↑
Task-specific functional training [36,37,38,46]	Commands followed; path clear; equipment ready	Repetitions/time ↑ → assistance ↓ → integrate standing/stepping → add dual-task
In-bed cycle ergometry (lower limbs) [50,81]	Stable oxygenation/hemodynamics; ventilator tolerated; lines secured	Assisted → active time ↑ → resistance/gear ↑ → rest intervals ↓
Upper-limb ergometer [31,49,67,69]	Stable SpO_2_/MAP; light sedation (RASS −2 to 0); line slack verified	Duration ↑ → cadence/resistance ↑ → assistance ↓
Bedside treadmill/body-weight support treadmill [51]	Harness fitted; team ready; orthostatic tolerance; device alarms tested	%body-weight support ↓ → speed/time ↑ → transition to overground
Robotic- or suspension-assisted [53,54]	Device compatibility; trained staff; airway/lines secured	Assistance/support ↓ → stepping duration/complexity ↑ → integrate conventional tasks

Abbreviations: EOB, edge of bed; SpO_2_, Peripheral Capillary Oxygen Saturation; MAP, mean arterial pressure. ↑, increase; ↓, decrease.

**Table 6:** Safety screening, in-session monitoring, and stop rules, typical thresholds.

Safety Domain	Pre-Session Screen	In-Session Monitoring	Stop Rules with Exercise
Oxygenation (FiO_2_/SpO_2_) [36,40,42,46,49,50]	FiO_2_ ≤ 0.6; SpO_2_ ≥ 90% *; no acute distress; lines secured.	SpO_2_ continuous; dyspnea/fatigue queried; pace/time adjusted as tolerated.	SpO_2_ < 88–90% or symptomatic drop → stop, seated rest, return to prior level, raise O_2_ per protocol, notify.
Ventilation setting (PEEP) [36,40,42,46,50,65]	PEEP ≤ 10–12; ventilator tolerated; airway secure; team ready.	Observe ventilator synchrony; RR/work of breathing checked.	Loss of synchrony or distress → stop, rest, reposition airway/lines, prior level on resumption.
Hemodynamics (MAP/HR) [36,40,42,65,66,82]	MAP ≥ 65 mmHg; no unstable arrhythmia; low-dose vasoactive permitted with enhanced monitoring.	HR/BP rhythm observed; symptoms queried.	MAP < 65 or symptomatic tachy/brady/arrhythmia → stop, seated rest, prior level on resumption, notify.
Sedation/Delirium (RASS/CAM-ICU) [36,46,68,69,83]	RASS −2~0; follows simple commands; CAM-ICU documented.	Arousal maintained; attention/behavior observed.	Agitation or reduced arousal → stop, calm environment, resume at lower level when stable.
Lines/tubes security [36,40,42,50,65,67]	Access/airway/drains secured; route cleared; device check complete.	Line slack and fixation rechecked at transitions.	Line/device traction, leak, alarm → stop, secure/replace, reassess before resuming.
In-session monitoring [36,42,46,49,50,65]	Monitors available; baseline recorded; team roles confirmed.	SpO_2_/HR/BP/ECG as available; Borg/symptoms every few minutes.	Any predefined trigger → stop, document event, revert to prior level, inform team.
Stop rules (composite) [36,42,46,49,50,65,66,67]	Thresholds known to team; documentation ready.	Triggers watched: hypoxemia, hemodynamic instability, device issues, neurologic change, and patient request.	Trigger → immediate stop, safety first, document type/severity/action, plan modified on restart.

Note: * For institutions with a chronic hypoxemia protocol, an SpO_2_ threshold of 88% may be applied for screening and in-session monitoring. Abbreviations: FiO_2_, Fraction of Inspired Oxygen; SpO_2_, Peripheral Capillary Oxygen Saturation; PEEP, Positive End-Expiratory Pressure; RR, Respiration Rate; MAP, Mean Arterial Pressure; HR, Heart Rate; BP, Blood Pressure; RASS, Richmond Agitation–Sedation Scale; CAM-ICU, Confusion Assessment Method for the Intensive Care Unit; ECG, Electrocardiogram.

**Table 7:** Documentation minimum dataset for Physical Therapists-led ICU therapeutic exercise and mobility.

EMR Field	Definition/Examples	Field Format	Documentation Timing	Quality Rule/Validation
Provider and team attendance [36,40,42,65,67,84]	PT identifier; co-attendance by RN/RT/MD recorded.	PT name/initials; multi-select for attendees; required.	Per session; confirm pre-session; final check at session end.	PT presence required; mismatch with orders flagged.
Date/time and session duration [36,42,49,50,68]	Clock start–stop times; total minutes.	Datetime start; datetime end; auto-calculated duration.	Completed at session end.	Start < end; duration > 0; extreme values flagged.
Pre-session safety parameters (FiO_2_/SpO_2_/PEEP/MAP/RASS) [36,42,50,65,67]	Baseline values recorded immediately before activity: FiO_2_, SpO_2_, PEEP, MAP, and RASS.	Numeric (FiO_2_, SpO_2_, PEEP, MAP); ordinal (RASS). Units/scale displayed in field labels.	Within 15 min pre-session.	Outside thresholds requires rationale; missing values flagged
Activity level (IMS or equivalent) and assistance/device used [33,36,42,62,67,70]	Highest level achieved; assistance grade; device used.	IMS 0–10; assistance level; device list; distance/steps.	At session end.	Internal consistency check (level vs. assistance/device)
Planned vs. delivered dose and progression criteria [36,37,49,50]	Intended vs. delivered frequency/intensity/time; progression applied.	Planned fields; delivered fields; yes/no progression; reason if no.	During session and at session end	Variance >20% requires reason; progression aligned with safety rules.
In-session monitoring and patient-reported symptoms [36,42,49,50]	SpO_2_/HR/BP readings; dyspnea/fatigue/pain ratings.	Numeric time-stamped entries; Borg 0–10; pain 0–10	Baseline, peak, end.	Predefined triggers documented with action; missing intervals flagged.

Abbreviations: PT, Physical Therapists; RN, Registered Nurse; RT, Respiratory Therapists; MD, Medical Doctors; FiO_2_, Fraction of Inspired O_2_; SpO_2_, Peripheral Capillary Oxygen Saturation; PEEP, Positive End-Expiratory Pressure; MAP, Mean Arterial Pressure; RASS, Richmond Agitation–Sedation Scale; IMS, ICU Mobility Scale.

## 4. Discussion

This scoping review structures the range of PT-led therapeutic exercise and mobility in adult ICUs and aligns definitions, dosing and progression, safety parameters, and a minimum documentation dataset. While scoping reviews primarily map available evidence, they are uniquely satisfying the objective of identifying heterogeneity in practice. In this review, the creation of unified definitions was achieved not by establishing new norms, but by synthesizing common components found across included studies to resolve terminological inconsistencies and support future replication. Early, structured mobilization integrated with light sedation and daily physical therapy was feasible and improved short-term function in selected mechanically ventilated patients [8]. A multicenter trial of active mobilization reported no between-group difference and emphasized the need for careful selection, graded progression of intensity, and safety monitoring rather than intensity targets alone [10]. However, dependence on these guidelines requires critical interpretation, as inconsistencies in outcomes remain. For instance, recent large-scale trials have reported neutral results regarding mortality or functional recovery, leading to controversy over the optimal timing and intensity of interventions. These conflicting findings highlight the limitations of current evidence, which often lacks standardized definitions of ‘dose’ and ‘fidelity.’ Therefore, blindly adopting individual study protocols without addressing these operational heterogeneities may limit reproducibility. This review attempts to mitigate such reliance by synthesizing a unified framework that prioritizes definitional clarity over varying individual protocols. The combined safety parameters and stop criteria complement existing recommendations and translate them to bedside checklists and actions [12]. Aligning progression algorithms with outcome measures and time points supports consistent measurement using validated tools [20], and the higher achieved mobility levels and adequate session time were associated with better functional status at ICU discharge [22]. This review provides a practical reference in concise tables and a codebook to support unit-level quality review and comparisons across ICU using shared indicators. It also identifies priorities for trials, including the relative contribution of dose components and longer-term outcomes after discharge, consistent with prior reports [85,86]. This structure supports safe delivery, consistent measurement at defined time points, and quality improvement across adult ICU. However, it is important to acknowledge that the underlying evidence reflects a broad spectrum of practices rather than a uniform standard. The proposed definitions and progression logic, therefore, serve as a conceptual framework to organize these diverse findings rather than a reflection of universally consistent practice.

This section outlines principles that connect a readiness screen with prespecified prescription and a graded progression from in-bed exercise to EOB activities, transitions, transfers, and ambulation, with pass or hold cues guiding each step. Sedation and ventilator practice should enable participation, consistent with guidance that aligns light sedation with nonpharmacologic mobility and treats immobility as a daily treatment target [1,2]. Dose is structured by FITT and adjusted to physiologic and symptom responses, with planned and delivered minutes and task content recorded. A daily mobilization time of ≥40 min was associated with better functional status at ICU discharge [87]. Early mobilization within the first 72 h is part of structured ICU care and is supported by defined safety parameters and graded implementation procedures [88]. Dose progression is strengthened when outcome measures and time points are prespecified using validated tools such as the IMS and FSS-ICU, which demonstrate validity and responsiveness in adults in ICU [22,89]. Evidence from implementation studies shows that standardized orders, team communication routines, and tracking of delivered dose improve execution of mobility recommendations, supporting use of a minimum EMR dataset and progression algorithms [90]. In ICU practice, a PT-led dosing and graded progression approach, aligned to a readiness screen and documented in a minimum EMR dataset, supports safe delivery, consistent measurement at defined time points, and comparability across ICUs for quality improvement.

A practical sequence integrates a readiness screen with in-session monitoring and prespecified stop rules to support safe PT-led mobility. Readiness criteria include hemodynamic stability, adequate oxygenation, and a light sedation target that allows participation. Typical thresholds align with our safety table, including MAP ≥ 65 mmHg, FiO_2_ ≤ 0.6, SpO_2_ ≥ 90% with allowance for 88% in chronic hypoxemia protocols, PEEP 10–12 cmH_2_O, and a RASS target of −2~0 with stable vasoactive support [12]. It must be noted, however, that these specific values represent a synthesized operational range rather than a universally established standard. The included studies demonstrated inconsistency in specific cut-offs (e.g., precise HR or BP limits), indicating that while a general safety framework exists, consensus on rigid parameters remains limited. Furthermore, it is crucial to recognize that these standard progression criteria may require modification for patients with complex clinical presentations, such as those requiring high ventilatory support, experiencing vasoactive instability, or presenting with fluctuating delirium. In such high-acuity scenarios, strict adherence to a standard protocol may be insufficient; therefore, advanced clinical judgment combined with multidisciplinary consultation is essential to determine the feasibility and modification of safety parameters. During activity, the ICU rehabilitation team monitors SpO_2_, RR, HR, BP, symptoms, and communication. If thresholds are crossed, the response includes pausing the task, returning to the prior step, repositioning, adjusting oxygen or ventilator settings as per the safety table, notifying the team, and confirming recovery. Prior guidance provided parameter categories and decision criteria for ventilated adults, and subsequent trials emphasized careful selection and vigilant monitoring when implementing active mobilization [10]. Adverse events during ICU mobilization were uncommon, occurring in less than three percent of sessions, which supports the feasibility of structured monitoring and management procedure [13]. The EMR records stop criteria, responses implemented, and recovery status using the minimum dataset to provide verifiable documentation and support unit-level quality review. Adopting shared thresholds with a pass or hold readiness screen and documenting real-time responses in the EMR supports safe progression, consistent decision-making, and unit-level quality improvement.

An EMR minimum dataset that records provider attribution, date and time, duration, pre-session safety status, activity level with assistance or device, planned and delivered dose with progression, and in-session monitoring with symptoms supports transparent documentation and unit-level review. Quantifying delivered mobility within routine documentation is feasible and complementary to instrumented monitoring, which supports the use of standardized EMR fields to summarize frequency, intensity parameters, and time on task in adult ICU [91]. Measurement should align with defined time points and use validated assessments with known interpretability, including the IMS, which demonstrated construct validity, responsiveness, and a published minimal important difference [21,92]. The FSS-ICU shows internal consistency and responsiveness and provides an estimated minimal important difference that supports interpretation and sample-size planning [22]. Additional instruments with supportive measurement properties include the PFIT scored and the Chelsea Critical Care Physical Assessment tool. These instruments demonstrated validity and responsiveness, and predictive validity at or after ICU discharge has been reported [93,94]. Minimal important difference or responsiveness thresholds have been proposed for other ICU mobility indices such as the Perme ICU Mobility Score, indicating progress toward clinically meaningful change values across assessments [95]. Finally, documentation-focused quality initiatives that make mobility goals and delivered dose in the chart have been associated with improved team communication and shorter LOS in ICU, supporting the role of structured EMR fields for comparability and unit-level quality review [96]. However, it is recognized that implementing this comprehensive dataset presents practical challenges, as many current EMR systems lack dedicated fields for specific variables like ‘assistance level’ or ‘planned versus delivered dose’ without significant system modification. To address these operational constraints, a phased adoption strategy is recommended. Initially, institutions may incorporate these elements into free-text templates or standardized phrases to establish workflow familiarity. Subsequently, as institutional infrastructure permits, these fields can be integrated into the EMR system to enable automated data extraction and unit-level quality monitoring. Combining a standardized EMR minimum dataset with validated outcome measures at defined time points supports transparent prescription, clinically meaningful change scores, and interdisciplinary comparison for quality improvement.

Implementation depends on PT-led prescription and progression, with team routines that align sedation practice and daily mobility goals to enable participation. Practical barriers commonly include staffing, equipment access, competing clinical priorities, ICU culture, and role clarity [97,98]. Programs that used protocolized mobility delivered by an interdisciplinary team reported shorter ICU and hospital length of stay, which supports structured orders, dose recording, and daily review [5]. Adverse events during mobilization were uncommon, supporting the use of shared thresholds, stop criteria, and recovery checks as part of routine safety monitoring [99]. Implementation strategies that improved adherence and team communication included standardized order sets, scheduled team huddles, and assessment with feedback [100]. This scoping review contributes PT-led operational definitions, dose and progression principles, safety thresholds, and a standardized EMR dataset that support consistent delivery and comparability across ICU. Strengths include an evidence base focused on adult ICU and implementable specifications for practice and documentation.

A primary limitation is the reliance on a single reviewer for screening and data charting, which deviates from the dual-reviewer approach recommended by JBI and PRISMA-ScR guidelines. This approach introduces potential risks of selection bias and data extraction errors, thereby compromising the methodological rigor typical of systematic reviews. While this constraint was inherent to the study design, measures were implemented to mitigate these risks. Specifically, a random 20 percent sample at both title/abstract and full-text stages was rechecked by the author after a predefined interval to ensure intra-rater reliability. Reporting of dose, fidelity procedures, and measurement schedules varied across studies, which constrained comparisons and precluded meta-analysis. The protocol was developed a priori but not registered, and the protocol and the PRISMA-ScR checklist are provided in the Supplementary Materials. Inclusion was restricted to adults in ICUs and to publications in English or Korean, which may limit generalizability. A significant limitation of the included literature is the high degree of heterogeneity in intervention protocols and the frequent absence of fidelity reporting. Furthermore, many included studies utilized observational or quality-improvement designs rather than randomized controlled trials, which limits the ability to draw causal inferences regarding the efficacy of specific dose parameters.

Future research should test dose components and sequences of progression in randomized or stepped-wedge designs, refine safety parameters for patients receiving higher levels of ventilatory or vasoactive support, evaluate the effect of a PT-led documentation minimum dataset on care quality, and extend follow-up to patient-centered outcomes after discharge. In clinical practice, integrating PT-led prescription and progression with shared safety thresholds and standardized documentation offers a practical route to consistent delivery, reliable measurement, and quality improvement across adult ICU.

## 5. Conclusions

This scoping review summarizes the available evidence regarding PT-led therapeutic exercise and mobility in adult ICUs. The findings indicate that operationalizing PT-led prescription with a readiness screen and graded activity levels is feasible and can be recorded in routine EMRs. The synthesized data suggest that adopting shared thresholds and stop rules facilitates consistent decision-making, while aligning progression with validated outcome measures supports clinical interpretation. The review highlights that consistent delivery appears to be associated with defined PT roles and team routines, although reporting variability remains a challenge. Future research is needed to rigorously test specific dose components and safety parameters for higher-acuity patients. In summary, the mapped evidence supports a structured and PT-led approach as a viable model for safe delivery and quality improvement in adult ICU.

## Data Availability

No new data were created.

## Supplementary Materials

The following supporting information can be downloaded at: https://www.mdpi.com/article/10.3390/jcm14248948/s1, Supplementary S1: PRISMA-ScR checklist; Supplementary S2: Intervention range and activity level for Physical Therapists-led ICU rehabilitation with readiness criteria, dose parameters, minimum staffing and roles, and equipment and safety measures; Supplementary S3: Documentation codebook for EMR fields, formats and units, timing, required status, and validation rules; Supplementary S4: Barriers to ICU rehabilitation and implementation strategies; strategy pairing offers actionable countermeasures aligned to PT-led practice. References [101,102,103,104,105,106] are cited in the supplementary materials.

## Abbreviations

The following abbreviations are used in this manuscript:

ICU	Intensive care units
PT	Physical Therapists
OT	Occupational Therapists
LOS	Length of stay
FITT	Frequency/Intensity/Type/Time
IMS	ICU Mobility Scale
FSS-ICU	Functional Status Score for the ICU
MRC	Medical Research Council
EMR	Electrical Medical Record
JBI	Joanna Briggs Institute
EOB	Edge of the bed
ROM	Range of motion
OOB	Out of bed
SpO_2_	Peripheral Capillary Oxygen Saturation
RASS	Richmond Agitation–Sedation Scale
ADL	Activities of daily living
PFIT	Physical Function ICU Test
RPE	Rating of perceived exertion
HR	Heart Rate
RPM	Repetitions per Minute
CPM	Continuous passive movement
BSWT	Body-weight support treadmill
NMES	Neuromuscular electrical stimulation
VR	Virtual reality
FiO_2_	Fraction of Inspired Oxygen
PEEP	Positive End-Expiratory Pressure
MAP	Mean Arterial Pressure
RN	Registered Nurse
MD	Medical Doctor
RT	Respiratory Therapist
ETT	Endotracheal Tube
6MWT	6-Minute Walk Test
MCID	Minimal clinically important difference
MDC	Minimally detectable change
RR	Respiration Rate
BP	Blood Pressure
CAM-ICU	Confusion Assessment Method for the Intensive Care Unit
ECG	Electrocardiogram

## References

[1] Devlin J.W., Skrobik Y., Gélinas C., Needham D.M., Slooter A.J.C., Pandharipande P.P., Watson P.L., Weinhouse G.L., Nunnally M.E., Rochwerg B. (2018). Clinical Practice Guidelines for the Prevention and Management of Pain, Agitation/Sedation, Delirium, Immobility, and Sleep Disruption in Adult Patients in the ICU. Crit. Care Med..

[2] Lewis K., Balas M.C., Stollings J.L., McNett M., Girard T.D., Chanques G., Kho M.E., Pandharipande P.P., Weinhouse G.L., Brummel N.E. (2025). A Focused Update to the Clinical Practice Guidelines for the Prevention and Management of Pain, Anxiety, Agitation/Sedation, Delirium, Immobility, and Sleep Disruption in Adult Patients in the ICU. Crit. Care Med..

[3] Brummel N.E., Girard T.D., Ely E.W., Pandharipande P.P., Morandi A., Hughes C.G., Graves A.J., Shintani A., Murphy E., Work B. (2014). Feasibility and Safety of Early Combined Cognitive and Physical Therapy for Critically Ill Medical and Surgical Patients: The Activity and Cognitive Therapy in ICU (ACT-ICU) Trial. Intensive Care Med..

[4] Bertschi D., Rotondo F., Waskowski J., Venetz P., Pfortmueller C.A., Schefold J.C. (2025). Post-Extubation Dysphagia in the ICU−a Narrative Review: Epidemiology, Mechanisms and Clinical Management (Update 2025). Crit. Care.

[5] Morris P.E., Goad A., Thompson C., Taylor K., Harry B., Passmore L., Ross A., Anderson L., Baker S., Sanchez M. (2008). Early Intensive Care Unit Mobility Therapy in the Treatment of Acute Respiratory Failure. Crit. Care Med..

[6] Engel H.J., Needham D.M., Morris P.E., Gropper M.A. (2013). ICU Early Mobilization: From Recommendation to Implementation at Three Medical Centers. Crit. Care Med..

[7] Lang J.K., Paykel M.S., Haines K.J., Hodgson C.L. (2020). Clinical Practice Guidelines for Early Mobilization in the ICU: A Systematic Review. Crit. Care Med..

[8] Schweickert W.D., Pohlman M.C., Pohlman A.S., Nigos C., Pawlik A.J., Esbrook C.L., Spears L., Miller M., Franczyk M., Deprizio D. (2009). Early Physical and Occupational Therapy in Mechanically Ventilated, Critically Ill Patients: A Randomised Controlled Trial. Lancet.

[9] Li Z., Peng X., Zhu B., Zhang Y., Xi X. (2013). Active Mobilization for Mechanically Ventilated Patients: A Systematic Review. Arch. Phys. Med. Rehabil..

[10] Hodgson C.L., Bailey M., Bellomo R., Brickell K., Broadley T., Buhr H., Gabbe B.J., Gould D.W., Harrold M., TEAM Study Investigators and the ANZICS Clinical Trials Group (2022). Early Active Mobilization during Mechanical Ventilation in the ICU. N. Engl. J. Med..

[11] Sosnowski K., Lin F., Chaboyer W., Ranse K., Heffernan A., Mitchell M. (2023). The Effect of the ABCDE/ABCDEF Bundle on Delirium, Functional Outcomes, and Quality of Life in Critically Ill Patients: A Systematic Review and Meta-Analysis. Int. J. Nurs. Stud..

[12] Hodgson C.L., Stiller K., Needham D.M., Tipping C.J., Harrold M., Baldwin C.E., Bradley S., Berney S., Caruana L.R., Elliott D. (2014). Expert Consensus and Recommendations on Safety Criteria for Active Mobilization of Mechanically Ventilated Critically Ill Adults. Crit. Care Lond. Engl..

[13] Paton M., Chan S., Neto A.S., Tipping C.J., Stratton A., Lane R., Romero L., Broadley T., Hodgson C.L. (2024). Association of Active Mobilisation Variables with Adverse Events and Mortality in Patients Requiring Mechanical Ventilation in the Intensive Care Unit: A Systematic Review and Meta-Analysis. Lancet Respir. Med..

[14] Mayer K.P., Joseph-Isang E., Robinson L.E., Parry S.M., Morris P.E., Neyra J.A. (2020). Safety and Feasibility of Physical Rehabilitation and Active Mobilization in Patients Requiring Continuous Renal Replacement Therapy: A Systematic Review. Crit. Care Med..

[15] Hoffmann T.C., Glasziou P.P., Boutron I., Milne R., Perera R., Moher D., Altman D.G., Barbour V., Macdonald H., Johnston M. (2014). Better Reporting of Interventions: Template for Intervention Description and Replication (TIDieR) Checklist and Guide. BMJ.

[16] Ogrinc G., Davies L., Goodman D., Batalden P., Davidoff F., Stevens D. (2016). SQUIRE 2.0 (Standards for QUality Improvement Reporting Excellence): Revised Publication Guidelines from a Detailed Consensus Process. BMJ Qual. Saf..

[17] Park Y.H., Ko R.-E., Kang D., Park J., Jeon K., Yang J.H., Park C.-M., Cho J., Park Y.S., Park H. (2020). Relationship between Use of Rehabilitation Resources and ICU Readmission and ER Visits in ICU Survivors: The Korean ICU National Data Study 2008-2015. J. Korean Med. Sci..

[18] McLaughlin K.H., Friedman M., Hoyer E.H., Kudchadkar S., Flanagan E., Klein L., Daley K., Lavezza A., Schechter N., Young D. (2023). The Johns Hopkins Activity and Mobility Promotion Program. J. Nurs. Care Qual..

[19] Hough C.L., Lieu B.K., Caldwell E.S. (2011). Manual Muscle Strength Testing of Critically Ill Patients: Feasibility and Interobserver Agreement. Crit. Care.

[20] Hodgson C., Needham D., Haines K., Bailey M., Ward A., Harrold M., Young P., Zanni J., Buhr H., Higgins A. (2014). Feasibility and Inter-Rater Reliability of the ICU Mobility Scale. Heart Lung.

[21] Tipping C.J., Bailey M.J., Bellomo R., Berney S., Buhr H., Denehy L., Harrold M., Holland A., Higgins A.M., Iwashyna T.J. (2016). The ICU Mobility Scale Has Construct and Predictive Validity and Is Responsive. A Multicenter Observational Study. Ann. Am. Thorac. Soc..

[22] Huang M., Chan K.S., Zanni J.M., Parry S.M., Neto S.-C.G.B., Neto J.A.A., da Silva V.Z.M., Kho M.E., Needham D.M. (2016). Functional Status Score for the ICU: An International Clinimetric Analysis of Validity, Responsiveness, and Minimal Important Difference. Crit. Care Med..

[23] Vanhorebeek I., Latronico N., Van den Berghe G. (2020). ICU-Acquired Weakness. Intensive Care Med..

[24] Brodsky M.B., Nollet J.L., Spronk P.E., González-Fernández M. (2020). Prevalence, Pathophysiology, Diagnostic Modalities, and Treatment Options for Dysphagia in Critically Ill Patients. Am. J. Phys. Med. Rehabil..

[25] da Conceição T.M.A., Gonzáles A.I., de Figueiredo F.C.X.S., Vieira D.S.R., Bündchen D.C. (2017). Safety criteria to start early mobilization in intensive care units. Systematic review. Rev. Bras. Ter. Intensiva.

[26] Tricco A.C., Lillie E., Zarin W., O’Brien K.K., Colquhoun H., Levac D., Moher D., Peters M.D.J., Horsley T., Weeks L. (2018). PRISMA Extension for Scoping Reviews (PRISMA-ScR): Checklist and Explanation. Ann. Intern. Med..

[27] Peters M.D.J., Marnie C., Tricco A.C., Pollock D., Munn Z., Alexander L., McInerney P., Godfrey C.M., Khalil H. (2020). Updated Methodological Guidance for the Conduct of Scoping Reviews. JBI Evid. Synth..

[28] Munn Z., Peters M.D.J., Stern C., Tufanaru C., McArthur A., Aromataris E. (2018). Systematic Review or Scoping Review? Guidance for Authors When Choosing between a Systematic or Scoping Review Approach. BMC Med. Res. Methodol..

[29] Rethlefsen M.L., Kirtley S., Waffenschmidt S., Ayala A.P., Moher D., Page M.J., Koffel J.B., PRISMA-S Group (2021). PRISMA-S: An Extension to the PRISMA Statement for Reporting Literature Searches in Systematic Reviews. Syst. Rev..

[30] McGowan J., Sampson M., Salzwedel D.M., Cogo E., Foerster V., Lefebvre C. (2016). PRESS Peer Review of Electronic Search Strategies: 2015 Guideline Statement. J. Clin. Epidemiol..

[31] Martí J.D., McWilliams D., Gimeno-Santos E. (2020). Physical Therapy and Rehabilitation in Chronic Obstructive Pulmonary Disease Patients Admitted to the Intensive Care Unit. Semin. Respir. Crit. Care Med..

[32] Jacob P., Gupta P., Shiju S., Omar A.S., Ansari S., Mathew G., Varghese M., Pulimoottil J., Varkey S., Mahinay M. (2021). Multidisciplinary, Early Mobility Approach to Enhance Functional Independence in Patients Admitted to a Cardiothoracic Intensive Care Unit: A Quality Improvement Programme. BMJ Open Qual..

[33] Suzuki G., Kanayama H., Arai Y., Iwanami Y., Kobori T., Masuyama Y., Yamamoto S., Serizawa H., Nakamichi Y., Watanabe M. (2024). Early Mobilization Using a Mobile Patient Lift in the ICU: A Randomized Controlled Trial. Crit. Care Med..

[34] Viloria M.A.D., Lee S.-D., Takahashi T., Cheng Y.-J. (2023). Physical Therapy in the Intensive Care Unit: A Cross-Sectional Study of Three Asian Countries. PLoS ONE.

[35] Uhlig S.E., Rodrigues M.K., Oliveira M.F., Tanaka C. (2024). Timing to Out-of-Bed Mobilization and Mobility Levels of COVID-19 Patients Admitted to the ICU: Experiences in Brazilian Clinical Practice. Physiother. Theory Pract..

[36] Ho L., Tsang J.H.C., Cheung E., Chan W.Y., Lee K.W., Lui S.R., Lee C.Y., Lee A.L.H., Lam P.K.N. (2022). Improving Mobility in the Intensive Care Unit with a Protocolized, Early Mobilization Program: Observations of a Single Center before-and-after the Implementation of a Multidisciplinary Program. Acute Crit. Care.

[37] Schaller S., Anstey M., Blobner M., Edrich T., Grabitz S., Gradwohl-Matis I., Heim M., Houle T., Kurth T., Latronico N. (2016). Early, Goal-Directed Mobilisation in the Surgical Intensive Care Unit: A Randomised Controlled Trial. Lancet.

[38] McWilliams D., Snelson C., Goddard H., Attwood B. (2019). Introducing Early and Structured Rehabilitation in Critical Care: A Quality Improvement Project. Intensive Crit. Care Nurs..

[39] de Paula M.A.S., Carvalho E.V., de Souza Vieira R., Bastos-Netto C., de Jesus L.A.D.S., Stohler C.G., Arantes G.C., Colugnati F.A.B., Reboredo M.M., Pinheiro B.V. (2024). Effect of a Structured Early Mobilization Protocol on the Level of Mobilization and Muscle Strength in Critical Care Patients: A Randomized Clinical Trial. Physiother. Theory Pract..

[40] Amundadottir O.R., Jónasdóttir R.J., Sigvaldason K., Gunnsteinsdottir E., Haraldsdottir B., Sveinsson T., Sigurdsson G.H., Dean E. (2021). Effects of Intensive Upright Mobilisation on Outcomes of Mechanically Ventilated Patients in the Intensive Care Unit: A Randomised Controlled Trial with 12-Months Follow-Up. Eur. J. Physiother..

[41] Schujmann D.S., Lunardi A.C., Fu C. (2018). Progressive Mobility Program and Technology to Increase the Level of Physical Activity and Its Benefits in Respiratory, Muscular System, and Functionality of ICU Patients: Study Protocol for a Randomized Controlled Trial. Trials.

[42] Sasano N., Kato Y., Tanaka A., Kusama N. (2022). Out-of-the-ICU Mobilization in Critically Ill Patients: The Safety of a New Model of Rehabilitation. Crit. Care Explor..

[43] Linke C.A., Chapman L.B., Berger L.J., Kelly T.L., Korpela C.A., Petty M.G. (2020). Early Mobilization in the ICU: A Collaborative, Integrated Approach. Crit. Care Explor..

[44] Laurent H., Aubreton S., Vallat A., Pereira B., Souweine B., Constantin J.-M., Coudeyre E. (2020). Very Early Exercise Tailored by Decisional Algorithm Helps Relieve Discomfort in ICU Patients: An Open-Label Pilot Study. Eur. J. Phys. Rehabil. Med..

[45] McGarrigle L., Caunt J. (2016). Physical Therapist-Led Ambulatory Rehabilitation for Patients Receiving CentriMag Short-Term Ventricular Assist Device Support: Retrospective Case Series. Phys. Ther..

[46] Brummel N., Jackson J., Girard T., Pandharipande P., Schiro E., Work B., Pun B., Boehm L., Gill T., Ely E. (2012). A Combined Early Cognitive and Physical Rehabilitation Program for People Who Are Critically Ill: The Activity and Cognitive Therapy in the Intensive Care Unit (ACT-ICU) Trial. Phys. Ther..

[47] Koester K., Troeller H., Panter S., Winter E., Patel J.J. (2018). Overview of Intensive Care Unit-Related Physical and Functional Impairments and Rehabilitation-Related Devices. Nutr. Clin. Pract..

[48] Weblin J., Harriman A., Butler K., Snelson C., McWilliams D. (2023). Comparing Rehabilitation Outcomes for Patients Admitted to the Intensive Care Unit with COVID-19 Requiring Mechanical Ventilation during the First Two Waves of the Pandemic: A Service Evaluation. Intensive Crit. Care Nurs..

[49] Hirakawa K., Nakayama A., Arimitsu T., Kon K., Ueki H., Hori K., Ishimoto Y., Ogawa A., Higuchi R., Hosoya Y. (2025). Feasibility and Safety of Upper Limb Extremity Ergometer Exercise in the Cardiac Intensive Care Unit in Critically Ill Patients with Cardiac Disease: A Prospective Observational Study. Front. Physiol..

[50] Kimawi I., Lamberjack B., Nelliot A., Toonstra A.L., Zanni J., Huang M., Mantheiy E., Kho M.E., Needham D.M. (2017). Safety and Feasibility of a Protocolized Approach to In-Bed Cycling Exercise in the Intensive Care Unit: Quality Improvement Project. Phys. Ther..

[51] Sommers J., Wieferink D.C., Dongelmans D.A., Nollet F., Engelbert R.H.H., van der Schaaf M. (2017). Body Weight-Supported Bedside Treadmill Training Facilitates Ambulation in ICU Patients: An Interventional Proof of Concept Study. J. Crit. Care.

[52] Kwakman R.C.H., Voorn E.L., Horn J., Nollet F., Engelbert R.H.H., Sommers J., van der Schaaf M. (2022). Steps to Recovery: Body Weight-Supported Treadmill Training for Critically Ill Patients: A Randomized Controlled Trial. J. Crit. Care.

[53] Lorenz M., Baum F., Kloss P., Langer N., Arsene V., Warner L., Panelli A., Hartmann F.V., Fuest K., Grunow J.J. (2024). Robotic-Assisted In-Bed Mobilization in Ventilated ICU Patients With COVID-19: An Interventional, Randomized, Controlled Pilot Study (ROBEM II Study). Crit. Care Med..

[54] Xu L., Wu H., Huang X., Song J., Fang F. (2025). Early Efficacy Observation of Suspended Lower-Limb Rehabilitation Robot-Assisted Therapy in Patients with Intensive Care Unit-Acquired Weakness: A Study Protocol for a Self-Controlled Randomised Controlled Trial. BMJ Open.

[55] Dos Santos F.V., Cipriano G.J., Vieira L., Güntzel Chiappa A.M., Cipriano G.B.F., Vieira P., Zago J.G., Castilhos M., da Silva M.L., Chiappa G.R. (2020). Neuromuscular Electrical Stimulation Combined with Exercise Decreases Duration of Mechanical Ventilation in ICU Patients: A Randomized Controlled Trial. Physiother. Theory Pract..

[56] Bao W., Yang J., Li M., Chen K., Ma Z., Bai Y., Xu Y. (2022). Prevention of Muscle Atrophy in ICU Patients without Nerve Injury by Neuromuscular Electrical Stimulation: A Randomized Controlled Study. BMC Musculoskelet. Disord..

[57] Othman S., Elbiaa M., Mansour E., El-Menshawy A., Elsayed S. (2024). Effect of Neuromuscular Electrical Stimulation and Early Physical Activity on ICU-Acquired Weakness in Mechanically Ventilated Patients: A Randomized Controlled Trial. Nurs. Crit. Care.

[58] Akar O., Günay E., Sarinc Ulasli S., Ulasli A.M., Kacar E., Sariaydin M., Solak Ö., Celik S., Ünlü M. (2017). Efficacy of Neuromuscular Electrical Stimulation in Patients with COPD Followed in Intensive Care Unit. Clin. Respir. J..

[59] Patsaki I., Gerovasili V., Sidiras G., Karatzanos E., Mitsiou G., Papadopoulos E., Christakou A., Routsi C., Kotanidou A., Nanas S. (2017). Effect of Neuromuscular Stimulation and Individualized Rehabilitation on Muscle Strength in Intensive Care Unit Survivors: A Randomized Trial. J. Crit. Care.

[60] Wang J., Shi C., Jia Y., Xiao Q. (2025). Effectiveness of Virtual Reality Assisted Active Limb Movement Exercises for Patients in the Respiratory Intensive Care Unit: A Randomized Pilot Study. J. Rehabil. Med..

[61] Brummel N., Jackson J., Girard T., Pandharipande P., Boehm L., Okahashi J., Strength C., Schiro E., Work B., Pun B. (2012). Feasibility of an Early Physical and Cognitive Rehabilitation Protocol for Critically Ill Patients: The Activity and Cognitive Therapy in the ICU (ACT-ICU) Trial. Am. J. Respir. Crit. Care Med..

[62] Hodgson C.L., Bailey M., Bellomo R., Berney S., Buhr H., Denehy L., Gabbe B., Harrold M., Higgins A., Iwashyna T.J. (2016). A Binational Multicenter Pilot Feasibility Randomized Controlled Trial of Early Goal-Directed Mobilization in the ICU. Crit. CARE Med..

[63] Holdsworth C., Haines K.J., Francis J.J., Marshall A., O’Connor D., Skinner E.H. (2015). Mobilization of Ventilated Patients in the Intensive Care Unit: An Elicitation Study Using the Theory of Planned Behavior. J. Crit. CARE.

[64] Tadyanemhandu C., Manie S. (2016). Implementation of the Physical Function ICU Test Tool in a Resource Constrained Intensive Care Unit to Promote Early Mobilisation of Critically Ill Patients—A Feasibility Study. Arch. Physiother..

[65] Nydahl P., Günther U., Diers A., Hesse S., Kerschensteiner C., Klarmann S., Borzikowsky C., Köpke S. (2020). PROtocol-Based MObilizaTION on Intensive Care Units: Stepped-Wedge, Cluster-Randomized Pilot Study (Pro-Motion). Nurs. Crit. Care.

[66] Katsukawa H., Ota K., Liu K., Morita Y., Watanabe S., Sato K., Ishii K., Yasumura D., Takahashi Y., Tani T. (2021). Risk Factors of Patient-Related Safety Events during Active Mobilization for Intubated Patients in Intensive Care Units-A Multi-Center Retrospective Observational Study. J. Clin. Med..

[67] Boyd J., Paratz J., Tronstad O., Caruana L., Walsh J. (2020). Exercise Is Feasible in Patients Receiving Vasoactive Medication in a Cardiac Surgical Intensive Care Unit: A Prospective Observational Study. Aust. Crit. Care.

[68] Parry S.M., Nydahl P., Needham D.M. (2018). Implementing Early Physical Rehabilitation and Mobilisation in the ICU: Institutional, Clinician, and Patient Considerations. Intensive Care Med..

[69] Hashem M.D., Nelliot A., Needham D.M. (2016). Early Mobilization and Rehabilitation in the ICU: Moving Back to the Future. Respir. Care.

[70] Escalon M.X., Lichtenstein A.H., Posner E., Spielman L., Delgado A., Kolakowsky-Hayner S.A. (2020). The Effects of Early Mobilization on Patients Requiring Extended Mechanical Ventilation Across Multiple ICUs. Crit. Care Explor..

[71] Saravankumar J., Paramaswamy R., Annadurai B., Iswarya S., Santhana Lakshmi S., Vishnuram S., Jeslin G.N., Sundaram Subramanian S., Senthilkumar N. (2024). Effect of Early Mobilization on Functional Recovery in ICU Patients with Post-COVID ARDS. Fizjoterapia Pol..

[72] Sakai Y., Taniuchi K., Karasawa T., Matsui K., Matsumoto T., Ikegami S., Imamura H., Horiuchi H. (2025). The Impact of Early Mobilization on the Incidence of Intensive Care Unit-Acquired Weakness in Patients with Sepsis in the Critical Care-The Shinshu Multicenter Prospective Cohort Study (EROSCCS Study). J. Clin. Med..

[73] Connolly B., Thompson A., Douiri A., Moxham J., Hart N. (2015). Exercise-Based Rehabilitation after Hospital Discharge for Survivors of Critical Illness with Intensive Care Unit-Acquired Weakness: A Pilot Feasibility Trial. J. Crit. Care.

[74] Wu D., Geng X., Wu H., Liu X., Liu X., Ma L., Li Y., Liang X., Lan Q., Wang Y. (2024). Effect of Early Mobilization on the Development of Pneumonia in Patients with Traumatic Brain Injury in the Neurosurgical Intensive Care Unit: A Historical Controls Study. Nurs. Crit. Care.

[75] Zhang C., Wang X., Mi J., Zhang Z., Luo X., Gan R., Mu S. (2024). Effects of the High-Intensity Early Mobilization on Long-Term Functional Status of Patients with Mechanical Ventilation in the Intensive Care Unit. Crit. Care Res. Pract..

[76] Shelly A.G., Prabhu N.S., Jirange P., Kamath A., Vaishali K. (2017). Quality of Life Improves with Individualized Home-Based Exercises in Critical Care Survivors. Indian J. Crit. Care Med..

[77] Johnson A.M., Henning A.N., Morris P.E., Tezanos A.G.V., Dupont-Versteegden E.E. (2017). Timing and Amount of Physical Therapy Treatment Are Associated with Length of Stay in the Cardiothoracic ICU. Sci. Rep..

[78] Zakeri M.A., Aziz A.R., Rahiminezhad E., Dehghan M. (2024). Effectiveness of Massage and Range of Motion Exercises on Muscle Strength and Intensive Care Unit-Acquired Weakness in Iranian Patients with COVID-19: A Randomized Parallel-Controlled Trial. Acute Crit. Care.

[79] de Campos Biazon T.M.P., Libardi C.A., Junior J.C.B., Caruso F.R., da Silva Destro T.R., Molina N.G., Borghi-Silva A., Mendes R.G. (2021). The Effect of Passive Mobilization Associated with Blood Flow Restriction and Combined with Electrical Stimulation on Cardiorespiratory Safety, Neuromuscular Adaptations, Physical Function, and Quality of Life in Comatose Patients in an ICU: A Randomized Controlled Clinical Trial. Trials.

[80] Ichikawa T., Tsuchiya A., Tsutsumi Y., Okawa T., Kubo D., Horimizu Y., Tsutsui R., Shukumine H., Noda K., Mizuno K. (2025). Effect of a Generalized Early Mobilization and Rehabilitation Protocol on Outcomes in Trauma Patients Admitted to the Intensive Care Unit: A Retrospective Pre–Post Study. Crit. Care.

[81] Sommers J., Van den Boorn M., Engelbert R.H.H., Nollet F., Van der Schaaf M., Horn J. (2018). Feasibility of Muscle Activity Assessment With Surface Electromyography During Bed Cycling Exercise In Intensive Care Unit Patients. Muscle Nerve.

[82] Nickels M.R., Blythe R., White N., Ali A., Aitken L.M., Heyland D.K., McPhail S.M. (2023). Predictors of Acute Muscle Loss in the Intensive Care Unit: A Secondary Analysis of an in-Bed Cycling Trial for Critically Ill Patients. Aust. Crit. Care.

[83] Garegnani L., Ivaldi D., Burgos M.A., Varela L.B., Díaz Menai S., Rico S., Giménez M.L., Escobar Liquitay C.M., Franco J.V. (2025). Exercise Therapy for the Treatment of Delirium in the Intensive Care Unit. Cochrane Database Syst. Rev..

[84] Wischmeyer P.E., Puthucheary Z., San Millán I., Butz D., Grocott M.P.W. (2017). Muscle Mass and Physical Recovery in ICU: Innovations for Targeting of Nutrition and Exercise. Curr. Opin. Crit. Care.

[85] Monsees J., Moore Z., Patton D., Watson C., Nugent L., Avsar P., O’Connor T. (2023). A Systematic Review of the Effect of Early Mobilisation on Length of Stay for Adults in the Intensive Care Unit. Nurs. Crit. Care.

[86] Matsuoka A., Yoshihiro S., Shida H., Aikawa G., Fujinami Y., Kawamura Y., Nakanishi N., Shimizu M., Watanabe S., Sugimoto K. (2023). Effects of Mobilization within 72 h of ICU Admission in Critically Ill Patients: An Updated Systematic Review and Meta-Analysis of Randomized Controlled Trials. J. Clin. Med..

[87] Lorenz M., Fuest K., Ulm B., Grunow J.J., Warner L., Bald A., Arsene V., Verfuß M., Daum N., Blobner M. (2023). The Optimal Dose of Mobilisation Therapy in the ICU: A Prospective Cohort Study. J. Intensive Care.

[88] Schaller S.J., Scheffenbichler F.T., Bein T., Blobner M., Grunow J.J., Hamsen U., Hermes C., Kaltwasser A., Lewald H., Nydahl P. (2024). Guideline on Positioning and Early Mobilisation in the Critically Ill by an Expert Panel. Intensive Care Med..

[89] Thrush A., Steenbergen E. (2022). Clinical Properties of the 6-Clicks and Functional Status Score for the ICU in a Hospital in the United Arab Emirates. Arch. Phys. Med. Rehabil..

[90] Hume N.E., Zerfas I., Wong A., Klein-Fedyshin M., Smithburger P.L., Buckley M.S., Devlin J.W., Kane-Gill S.L. (2024). Clinical Impact of the Implementation Strategies Used to Apply the 2013 Pain, Agitation/Sedation, Delirium or 2018 Pain, Agitation/Sedation, Delirium, Immobility, Sleep Disruption Guideline Recommendations: A Systematic Review and Meta-Analysis. Crit. Care Med..

[91] Fazio S., Doroy A., Da Marto N., Taylor S., Anderson N., Young H.M., Adams J.Y. (2020). Quantifying Mobility in the ICU: Comparison of Electronic Health Record Documentation and Accelerometer-Based Sensors to Clinician-Annotated Video. Crit. Care Explor..

[92] Tipping C.J., Holland A.E., Harrold M., Crawford T., Halliburton N., Hodgson C.L. (2018). The Minimal Important Difference of the ICU Mobility Scale. Heart Lung.

[93] Denehy L., de Morton N., Skinner E., Edbrooke L., Haines K., Warrillow S., Berney S. (2013). A Physical Function Test for Use in the Intensive Care Unit: Validity, Responsiveness, and Predictive Utility of the Physical Function ICU Test (Scored). Phys. Ther..

[94] Corner E.J., Soni N., Handy J.M., Brett S.J. (2014). Construct Validity of the Chelsea Critical Care Physical Assessment Tool: An Observational Study of Recovery from Critical Illness. Crit. Care Lond. Engl..

[95] Kenji Nawa R., Luiz Ferreira De Camillis M., Buttignol M., Machado Kutchak F., Chaves Pacheco E., Rodrigues Gonçalves L.H., Correa Garcia L.M., Tavares Timenetsky K., Forgiarini L.A. (2023). Clinimetric Properties of the Perme Intensive Care Unit Mobility Score—A Multicenter Study for Minimum Important Difference and Responsiveness Analysis. Colomb. Medica Cali Colomb..

[96] Anderson R.J., Sparbel K., Barr R.N., Doerschug K., Corbridge S. (2018). Electronic Health Record Tool to Promote Team Communication and Early Patient Mobility in the Intensive Care Unit. Crit. Care Nurse.

[97] Barber E.A., Everard T., Holland A.E., Tipping C., Bradley S.J., Hodgson C.L. (2015). Barriers and Facilitators to Early Mobilisation in Intensive Care: A Qualitative Study. Aust. Crit. Care.

[98] Bennion J., Manning C., Mansell S.K., Garrett R., Martin D. (2024). The Barriers to and Facilitators of Implementing Early Mobilisation for Patients with Delirium on Intensive Care Units: A Systematic Review. J. Intensive Care Soc..

[99] Nydahl P., Sricharoenchai T., Chandra S., Kundt F.S., Huang M., Fischill M., Needham D.M. (2017). Safety of Patient Mobilization and Rehabilitation in the Intensive Care Unit. Systematic Review with Meta-Analysis. Ann. Am. Thorac. Soc..

[100] Pun B.T., Balas M.C., Barnes-Daly M.A., Thompson J.L., Aldrich J.M., Barr J., Byrum D., Carson S.S., Devlin J.W., Engel H.J. (2019). Caring for Critically Ill Patients with the ABCDEF Bundle: Results of the ICU Liberation Collaborative in Over 15,000 Adults. Crit. Care Med..

[101] Cambiaso-Daniel J., Parry I., Rivas E., Kemp-Offenberg J., Sen S., Rizzo J.A., Serghiou M.A., Kowalske K., Wolf S.E., Herndon D.N. (2018). Strength and Cardiorespiratory Exercise Rehabilitation for Severely Burned Patients During Intensive Care Units: A Survey of Practice. J. Burn Care Res..

[102] Ferre M., Batista E., Solanas A., Martinez-Balleste A. (2021). Smart Health-Enhanced Early Mobilisation in Intensive Care Units. Sensors.

[103] Nickels M.R., Aitken L.M., Walsham J., Crampton L.J., Barnett A.G., McPhail S.M. (2020). Exercise Interventions Are Delayed in Critically Ill Patients: A Cohort Study in an Australian Tertiary Intensive Care Unit. Physiotherapy.

[104] Korupolu R., Zanni J.M., Fan E., Butler M., Needham D.M. (2010). Early Mobilisation of Intensive Care Unit Patient: The Challenges of Morbid Obesity and Multiorgan Failure. BMJ Case Rep..

[105] Roberts M., Johnson L., Lalonde T. (2014). Early Mobility in the Intensive Care Unit: Standard Equipment vs a Mobility Platform. Am. J. Crit. Care.

[106] Akhtar P.M., Deshmukh P.K. (2021). Knowledge, Attitudes, and Perceived Barriers of Healthcare Providers toward Early Mobilization of Adult Critically Ill Patients in Intensive Care Unit. Indian J. Crit. Care Med..

