# Supplementary material for: Physical Therapist-Led Therapeutic Exercise and Mobility in Adult Intensive Care Units: A Scoping Review of Operational Definitions, Dose Progression, Safety, and Documentation
Source: J Clin Med. 2025 Dec 18;14(24):8948. doi: 10.3390/jcm14248948 (PMC12733592; doi:10.3390/jcm14248948)
Supplement: Supplementary file 1 [file jcm-14-08948-s001.zip › jcm-4035585-supplementary S2~S4_Revised.pdf]

Supplement S2. Intervention range and activity ladder for PT-led ICU rehabilitation (coverage, parameters, staffing, equipment).

Activity level	Readiness criteria	Typical dose parameters	Minimum staffing & roles	Equipment & safeguards
In-bed (level 1) [31,36,37,50,53,65,101]	FiO_2 ≤0.6; SpO_2 ≥90%*; PEEP ≤10-12; MAP ≥65; RASS -2~0; lines secure.	1-2 sessions/day; 10-20 min; light effort; progress time → resistance.	PT leads; RN assists for lines; RT available if ventilated.	In-bed cycle or simple aids; portable monitor; line slack planned.
Edge of bed (level 2) [32,35–38,42]	Orthostatic tolerance acceptable; airway and lines secure; team ready.	1-2 sessions/day; 5-15 min sitting; progress duration → task challenge.	PT leads; spotter for trunk; RN manages lines.	Gait belt; non-slip surface; chair height adjusted.
Transition (level 3) [32,33,37,40,42]	Stable hemodynamics; follows commands; footwear safe; chair brakes locked.	3-5 attempts; 10-60 s stands; progress repetitions → stand time.	PT leads; second staff as spotter; RN oversees lines.	Gait belt; walker as needed; portable monitor; chair behind patient.
Transfer (level 4) [32,33,36–38,42]	Route clear; line slack planned; team positions assigned.	1-3 transfers/session; rest as needed; slide/pivot → stand-step.	PT leads; assistant for device/lines; RN confirms airway.	Chair locked and height set; walker or slide board if needed.
Ambulation (level 5) [36,37,40,42,62]	Orthostatic tolerance; portable monitoring; device management plan ready.	5–20 min walking; distance or pace ↑; rests ↓	PT leads; assistant manages lines/device; RT available if ventilated.	Walker or harness if needed; route pre-checked; alarms verified.
Across levels [36,40,42,46,65–67,102]	Baseline values recorded; thresholds known to team; roles confirmed.	Time ↑; → intensity ↑; → assistance ↓; → level ↑	PT leads within multidisciplinary team; staffing scaled to risk.	Line and tube check at each transition; fall-prevention plan; documentation ready.

Note: *For institutions with a chronic hypoxemia protocol, an SpO_2 threshold of 88% may be applied for screening and in-session monitoring; document the protocol and any deviations. **Abbreviations**: FiO_2, Fraction of Inspired O_2; SpO_2, Peripheral Capillary Oxygen Saturation; PEEP; Positive End-Expiratory Pressure; MAP, Mean Arterial Pressure; RASS, Richmond Agitation-Sedation Scale; PT, Physical Therapist; RN, Registered Nurse; RT, Respiratory Therapist.

Supplement S3. Documentation codebook for EMR capture, field definitions, validation rules, and coverage.

Field label	Definition/examples	Field format & units/scale	Documentation timing	Required	Validation rules/allowed values
Provider & team attendance [36,40,42,46,65–67]	PT identifier; co-attendance by RN/RT/MD	PT name or initials; multi-select for attendees	Session starts and end	Yes	PT must be present; attendee list from directory
Date/time & session duration [36,37,46,49,50,65]	Clock start-stop; total minutes	Datetime start; datetime end; auto duration (min)	Finalize at session end	Yes	Start < end; duration > 0; outliers flagged
Pre-session safety parameters (FiO_2/SpO_2/PEEP/MAP/RASS) [42,46,49,50,65,67]	Baseline oxygenation, ventilation, hemodynamics, sedation	Numeric (FiO_2, SpO_2, PEEP, MAP); ordinal (RASS)	Within 15 minutes pre-session	Yes	Thresholds per safety table; missing values flagged
Activity level (IMS or equivalent) & assistance/device used [33,36,40,42,51,62]	Highest level achieved; assistance grade; device	IMS 0-10; assistance scale; device dropdown	Session end	Yes	Internal consistency check level vs. assistance/device
Planned vs delivered dose & progression criteria [36,37,44,46,49–51,65]	Intended vs delivered FITT; progression applied	planned fields; delivered fields; yes/no progression	During and end of session	Yes	Variance > 20% requires reason; progression aligns with safety
In-session monitoring & symptoms [36,42,46,49,50,66,67]	SpO_2/HR/BP readings; dyspnea/fatigue/pain ratings	Time-stamped numeric; Borg 0–10; pain 0–10	Baseline, peak, end	Yes	Triggers documented with action; missing intervals flagged

Note: Validation rules are suggested for audit readiness; harmonize units/labels with Table 6 and indicators with Table 7.

Abbreviations: PT, Physical Therapist; RN, Registered Nurse; RT, Respiratory therapist; MD, Medical Doctor; FiO_2, Fraction of Inspired oxygen; SpO_2, Peripheral Capillary Oxygen Saturation; PEEP; Positive End-Expiratory Pressure; MAP, Mean Arterial Pressure; RASS, Richmond Agitation-Sedation Scale; IMS, ICU Mobility Scale; FITT, Frequency, Intensity, Type, Time; HR, Heart Rate; BP, Blood Pressure.

Supplement S4. Barriers to ICU rehabilitation and implementation strategies, strategy pairing offers actionable countermeasures aligned to physical therapists-led practice.

Barrier category	Physical therapists-led strategy	Key components & resources	Outcome measure & status
Patient instability and delirium [35,36,46,65–67,103]	Screen daily; dose within tolerance; integrate cognitive cues	Standardized screen; light sedation targets; delirium tools; staff education	Eligible days completed; holds with reasons; adverse events logged
Device burden and risk of traction/dislodgement during mobility [33,36,38,50,51,65,66,104]	Line plan before movement; route clear; secure and re-check at transitions	Checklists; fixation kits; portable monitoring; trained spotter	Device incidents; unplanned disconnections; completion rate of line checks
Sedation status [36,46,65,68,69,105]	Light sedation targets; mobility orders linked to sedation goals	Unit targets; RASS audit; nurse education; co-rounding with PT	Time in light sedation; mobility on sedation days; exceptions documented
Staffing and training [32,33,36,38,42,51,62,67,106]	Schedule PT-led sessions; competency ladder; just-in-time coaching	Staffing grid; skills checklist; simulation maneuvers	Sessions per eligible patient-day; competencies current; cancellations due to staffing
Medical leadership [32,35,36,38,65,103]	Fixed mobility with PT-triggered daily readiness screen; standing order at ICU admission to coordinate around procedures	Leadership endorsement; dashboard access; policy memo	Physician sign-off rate; order activation time; round-day mobility rate
Protocols and orders [32,33,38,49–51]	Clear readiness criteria; default order set; stepwise progression rules	Order set in EMR; pocket card; laminated flow sheet	Order uptake; protocol deviations with rationale; re-start within 24 h

Equipment and space [32,33,38,49–51]	MAP device availability; mobile carts; staged storage near beds	Inventory list; maintenance schedule; loan pool	Device-related delays; time to equipment; utilization rate
